# An Evaluation of MEMS-IMU Performance on the Absolute Trajectory Error of Visual-Inertial Navigation System

**DOI:** 10.3390/mi13040602

**Published:** 2022-04-12

**Authors:** Yunfei Liu, Zhitian Li, Shuaikang Zheng, Pengcheng Cai, Xudong Zou

**Affiliations:** 1 State Key Laboratory of Transducer Technology, Aerospace Information Research Institute, Chinese Academy of Sciences, Beijing 100190, China; liuyunfei17@mails.ucas.ac.cn (Y.L.); ztli@mail.ie.ac.cn (Z.L.); zhengshuaikang18@mails.ucas.ac.cn (S.Z.); caipengcheng16@mails.ucas.ac.cn (P.C.); 2 School of Electronic, Electrical and Communication Engineering, University of Chinese Academy of Sciences, Beijing 100049, China

**Keywords:** MEMS-IMU, visual-inertial odometry, sensor fusion, MEMS applications

## Abstract

Nowadays, accurate and robust localization is preliminary for achieving a high autonomy for robots and emerging applications. More and more, sensors are fused to guarantee these requirements. A lot of related work has been developed, such as visual-inertial odometry (VIO). In this research, benefiting from the complementary sensing capabilities of IMU and cameras, many problems have been solved. However, few of them pay attention to the impact of different performance IMU on the accuracy of sensor fusion. When faced with actual scenarios, especially in the case of massive hardware deployment, there is the question of how to choose an IMU appropriately? In this paper, we chose six representative IMUs with different performances from consumer-grade to tactical grade for exploring. According to the final performance of VIO based on different IMUs in different scenarios, we analyzed the absolute trajectory error of Visual-Inertial Systems (VINS_Fusion). The assistance of IMU can improve the accuracy of multi-sensor fusion, but the improvement of fusion accuracy with different grade MEMS-IMU is not very significant in the eight experimental scenarios; the consumer-grade IMU can also have an excellent result. In addition, the IMU with low noise is more versatile and stable in various scenarios. The results build the route for the development of Inertial Navigation System (INS) fusion with visual odometry and at the same time, provide a guideline for the selection of IMU.

## 1. Introduction

In recent years, with the rapid growth in the fields of autonomous driving [1], augmented reality [2], virtual reality [3], and other emerging applications, the question of how to accurately obtain their localization information has become a crucial premise and foundation. To fulfill the requirements of these applications, a lot of exploration and novel work has been carried out by researchers, such as the work which fusions WiFi and IMU with floorplan [4]. Among many localization and navigation methods, inertial navigation is one of the mainstream methods at present. Inertial Measurement Unit (IMU) is the prerequisite of inertial navigation, as it plays a considerable role in indoor, urban high buildings, planetary exploration, and other GPS denial scenes. In terms of the indoor positioning technologies which heavily rely on IMU and other modal sensors, there are two major categories: building independent and dependent. Building independent draws support from image-based technologies and dead reckoning [5]. Building-dependent localization is realized by multi-modal sensors, such as Wi-Fi, Bluetooth, Ultra-Wide Band, Visible Light Communication, etc. With the development of the diversity of positioning technology, the hybrid patterns of indoor positioning based on smartphone cameras and IMU are also emerging [6]. IMU is significant for positioning and orientation applications. IMU is a device composed of a triaxial accelerometer that senses the linear acceleration coupled with the gravity of the body and a triaxial gyroscope that senses the angular velocity. The three axes are orthogonal to each other. Inevitably, due to the IMU being corrupted by inherent factors such as bias, noise, and random walk, localization is more and more unreliable; it is more exacerbated in low-cost IMU.

Thanks to the rapid improvement of the computing power of the platform, vision-based positioning methods have become more and more mature, such as visual odometry [7] which means that when a robot is in an unknown environment, this method can estimate its attitude by using the information of image while moving and exploring at the same time. In addition, the continuous breakthrough of deep learning boosts another new trend for the visual odometer, such as DeepVO [8] which is based on deep learning, and RoadMap [1] which is based on the semantic map. The end-to-end manner increases the adaptability and robustness of the scene. In the method of visual odometry, it is gratifying that the cumulative drift of trajectory is less than the inertial method, and the long-term stability is better than the inertial based. It is well known that the camera is used as an exteroceptive sensor for passive localization and features rich scene information. However, it is easy to be defeated in a dynamic environment, weak texture, fast motion, drastic changes in lighting and other scenes, and monocular visual odometry does not have distance perception. As an interoceptive sensor, IMU actively locates and is not susceptible to the environment. However, it is easily affected by noise, bias, and other inherent factors. How to fuse cameras and inertial or more information has become a research hotspot. Up to now, researchers have solved many problems by fusion camera and IMU, and many visual-inertial odometry (VIO) algorithms have been developed [9,10,11,12,13,14,15,16,17,18,19,20]. There are different schemes to fuse cameras and IMU which can be broadly categorized into the loosely-coupled [18,19,20] and the tightly-coupled [10,11,12,13,14,15,16,17]. VIO is also broadly divided into filtering-based [12,14,15] and optimization-based [10,11,13,16,17] in state estimation algorithm. The approaches based on optimization and tightly-coupled have more potential for accuracy [21].

**Figure 1:**
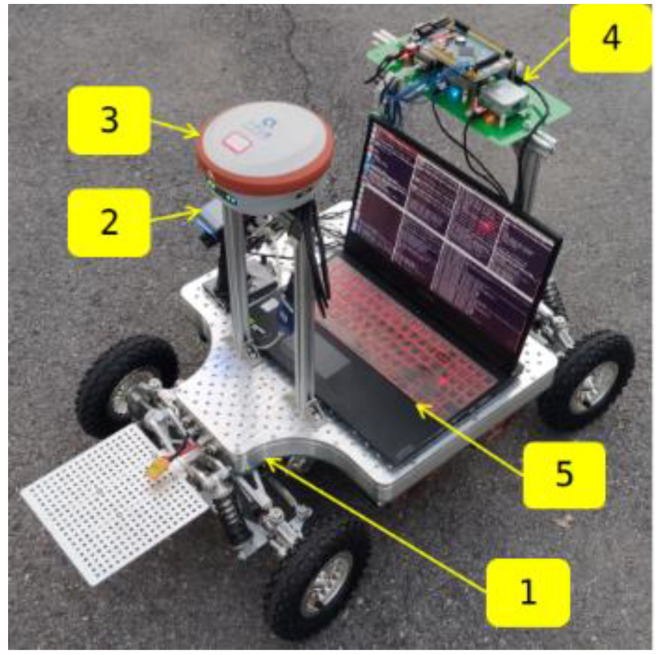
Platform setup. ➀ Wheeled Vehicle; ➁ Remote Control; ➂ Ground Truth C; ➃ Multi-IMU & Camera Suite; ➄ Laptop with Intel Core i7-9750H @2.60 GHz × 12.

As mentioned above, VIO-related algorithms have been rapidly developed. Nevertheless, few of them pay attention to the impact of different performance IMU on the accuracy of sensor fusion. What are the requirements for IMU performance in different situations? What is the impact of IMU with different performances on the accuracy of multi-sensor fusion especially for the emerging visual odometry technology? The analysis of this problem is significant to the hardware configuration, deployment of multi-sensor fusion systems, the development of IMU-based fusion localization technology, and other issues. To make it clearer, we chose the tightly-coupled algorithm VINS_Fusion [10,11] as the evaluation framework because of the representative in the operation based on sliding window optimization. Our platform is a wheeled robot running on the actual road, as shown in Figure 1. The main contributions in this study are summarized below. 

**Figure 2:**
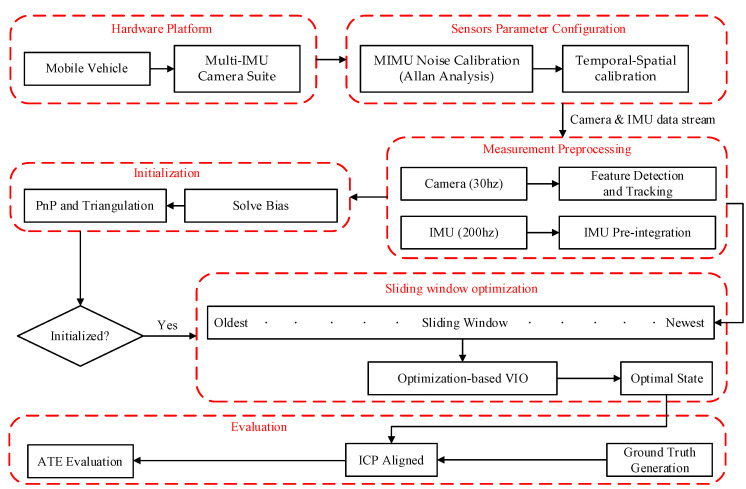
The framework for evaluation of MEMS-IMU performance on Visual-Inertial Navigation System.

In this paper, we propose a comparative evaluation framework to test the impact of different grade MEMS-IMU on the accuracy of the VIO algorithm as shown in Figure 2. In this paper, eight different experiments were designed, and a comprehensive evaluation was analyzed based on the absolute trajectory error. For a wide range of researchers, our experimental results are informative for sensor configuration and algorithms, and can clearly show the specific performance of different grade MEMS-IMU in VIO fusion accuracy. We can expediently select a MEMS-IMU for specific scenarios.

The rest of the paper is structured as follows: In Section 2, the related work about evaluation and analysis experiments and IMU selection are discussed. In Section 3, the framework flow of the overall experiment which is divided into four parts: hardware platforms (A), sensor setup (B), sensors parameter configuration (C), and evaluation (D) is presented. In Section 4, the results of the experiment are analyzed and discussed. Finally, the paper is concluded in Section 5. In addition, we append the absolute trajectory error of multiple IMUs in Section Appendix A.

## 2. Related Work

In order to make the positioning technologies more suitable for deployment and wide use, a lot of related work has been proposed. In the subsection Evaluation and Analysis Work, they paid more attention to the performance of many open-sourced algorithms on CPU/GPU. In subsection Multi-IMUs Work, the work on multiple IMUs is summarized, but their IMU type is single and the grade span is not wide. They mainly use redundant sensor groups to enhance the stability of the system. In addition, in partial work, MEMS-IMU was evaluated for user convenience.

### 2.1. Evaluation and Analysis Work

Six monocular visual-inertial algorithms were compared on several computing-constrained, single-board computers, and the accuracy of each algorithm, the time consumed per frame, and the utilization of CPU and memory were evaluated [22]. Similarly, Jinwoo et al. [23] compared the performance of nine visual(-inertial) odometry algorithms on different hardware platforms (Jetson TX2, Xavier NX, and AGX Xavier), evaluated the CPU utilization, and corresponding pose accuracy of each algorithm on each platform. Giubilato et al. [24] evaluated the performance of the visual odometry algorithm on Jetson TX2 and revealed the robustness, CPU/GPU utilization, frame rate, and so on. Alwin et al. [25] comprehensively evaluated and analyzed the performance of five sensor fusion algorithms of heading estimation using smartphone sensors, such as LKF, EKF, UKF, PF, and CF. These works are important for the selection of embedded platforms, fusion performance, and software deployment. However, they are more aimed at comparing the computing hardware and algorithms without considering the performance of the algorithms with different IMU. The impact of IMU with different performances on the accuracy of VIO is still blank.

### 2.2. Multi-IMUs Work

Three different IMU sensors were considered in [26,27], but they were mainly used to improve the robustness of the state estimation. Kevin et al. [27] utilized the information from multiple inertial measurement units in order to resile when one of them has sensor failures. Without considering the impact of different levels of IMU on the system, the authors in [28] exploited four IMUs and magnetometers with different positions to calculate the angular velocity and velocity information. However, there is no more consideration for the performance of IMU. In [29], Chao conducted a comparative investigation and evaluation on some low-cost IMUs, but focused on the sensor packages and available software solutions, and listed their specifications. The authors in [30] evaluated the accuracy of the IMU in the two smartphones and the inertial sensor in XIMU at a yaw angle. The IMU in the smartphone is MPU6500 and the IMU module is produced by BOSCH. XIMU refers to the combination of IMU and AHRS. The specific model is not indicated. The two IMUs used for comparison in this work are consumer IMUs, and the IMU with different performance is not considered. In [31], the authors evaluated four orientation algorithms (Madgwick, Mahony, EKF, TKF-Q) in the simulation environment. Nevertheless, this work was carried out in the simulation environment, considering the noise and bias instability, the veritable IMU will be affected by many factors, which cannot be represented in the simulation environment, and these IMUs were not evaluated in the actual environment. In [32], the authors carried out the evaluation of ZUPT, ZARU, and HDR algorithms which focus on heading drift reduction in pedestrian indoor navigation with consumption level IMU. In this work, the author focused on the error of the algorithm and did not analyze the impact of different performance IMUs. In [33], the authors evaluated seven commercial IMUs for persistent healthcare-related use cases. Through the analysis of the operating environment, cost performance, battery life, memory size, and other specifications, the selection criteria of IMU are obtained. This work considers more the convenience of these modules in use, and the performance span of different IMUs is relatively small. They are all assessed with consumer IMUs.

Compared with the above-related work, they did not pay attention to the grade, diversity of MEMS-IMU, and the impact of IMU on VIO fusion algorithms. In this paper, we have chosen six representative IMUs with different performances from consumer-grade to tactical grade for exploration and analysis.

## 3. Experiments and Methods

The specific framework of the experiment is shown in Figure 2. The hardware platform was built as shown in Figure 2 first, including the mobile platform and multi-IMU camera suite. Then, the sensors parameter configuration was conducted, including Allan’s analysis of variance, temporal-spatial calibration between IMU and camera, etc. Next, the algorithm was executed in the specified scene by controlling the hardware platform while recording the RTK (Real-Time Kinematic) data as the ground truth at the same time. The localization accuracy of RTK is ±(8 + 1 × 10^−6^ D) mm.

In order to explore the influence of IMU on sensor fusion accuracy, five kinds of motion states were designed: slow, normal, fast, varying velocity, and spin move. In these scenes, IMU has different levels of excitation to identify the IMU’s ability. For example, in uniform velocity scenes, IMU’s incentive is small. However, IMU will be more motivated in varying velocity and spin move scenes. At the same time, experiments in the strong light environment with different motion states and weak texture special scenes which to analyze the auxiliary effect of IMU were also carried out. In addition, the performance of the fusion system with different IMUs under long-term running conditions that last for 30 min was also explored. The specific scenes are shown in Table 1. The tightly-coupled algorithm about Visual-Inertial Systems (VINS_Fusion [10,11]) was executed as the evaluation framework. Firstly, feature detection [34] and tracking were carried out by leveraging visual information. By preintegrating [35,36] the IMU information, the motion constraints were built with regard to the timestamps of adjacent image frames. Then, the initialization procedure was invoked by using feature information and preintegration information, in order to maintain a constant amount of computing, the optimization algorithm based on the sliding window was adopted. Finally, because the two types of trajectories did not belong to the same coordinate, the alignment was carried out by an iterative closest point algorithm (ICP) [37] and evaluated the absolute trajectory error (ATE) of the corresponding experiment.

The ATE can be calculated by comparing the ground-truth value with the visual-inertial odometry results. $p_{i}^{rtk}$ represents the true position value of timestamp i, $p_{i}^{odo}$ expresses the position output of VIO, and $E_{i}$ represents the ATE. As shown in the section Appendix A, the RMSE, median, and mean statistical errors are calculated with ATE. Through the rigid body transformation S composed of R and t, we can align the estimated results to the real value. To solve S, we aligned the estimated trajectory with the RTK trajectory using ICP [37].

(1) $$E_{i}={\left(p_{i}^{rtk}\right)}^{-1}S\left(p_{i}^{odo}\right)$$

(2) $$RMSE\left(E_{1:n}\right)={\left(\frac{1}{n}\overset{n}{\underset{i=1}{\sum }}\|E_{i}\|{}^{2}\right)}^{1/2}$$

(3) $$\underset{R,t}{\min}J=\frac{1}{2}\sum_{i=1}^{k}{\left\|(p_{i}^{rtk}-\left(Rp_{i}^{odo}+t\right)\;)\right\|}_{2}^{2}$$

There is little drift for a period of time after the system starts executing, and the initial trajectory segment is used for alignment. If the whole trajectory is used for alignment, the error after a long time will be adjusted due to the transformation matrix S, giving rise to inconsistency error. Here, our trajectory segment selects the first 70 data (k=70) of the whole trajectory, that is, data of the first 15 s. In the experiment, because the vehicle ran on an approximate two-dimensional plane, we concentrated on the localization accuracy in x, y  directions. In a section of Appendix A, we summarized the comparison table of algorithm errors in different scenarios.

### 3.1. Fusion Algorithm of VIO

#### 3.1.1. System States

The vector states of the system include  n+1 pose state x  in the sliding window and m+1 landmarks, where n  represents the size of a sliding window.

(4) $$X_{system}=\left[x_{0},x_{1},\cdots ,x_{n},\lambda_{0},\lambda_{1},\cdots ,\lambda_{m}\right]$$

The pose state $x_{k}$ includes position, velocity, orientation, accelerometer bias, and gyroscope bias.

(5) $$x_{k}=\left[p_{wb_{k}},v_{wb_{k}},q_{wb_{k}},b_{a},b_{g}\right]$$

${\left(\cdot \right)}_{wb_{k}}$ represents the state of body frame b with respect to the world frame w at time k. $b_{a}$ and $b_{g}$ represent accelerometer bias and gyroscope bias respectively.

#### 3.1.2. Visual Constraints

Using the extracted feature points, we can construct visual constraints. According to the reprojection process of the camera, we can construct the error cost function, which is called the reprojection error, and the error is expressed as the estimated value minus the measured value:

(6) $$r_{visual}=\left[\begin{array}{c} \frac{x_{c_{j}}}{z_{c_{j}}}-u_{c_{j}}\\ \frac{y_{c_{j}}}{z_{c_{j}}}-v_{c_{j}} \end{array}\right]$$

The ${\left[\begin{array}{ccc} x_{c_{j}} & y_{c_{j}} & z_{c_{j}} \end{array}\right]}^{T}$ represents the estimated value of the feature points of frame ith projected to the jth frame camera coordinate system according to the transformation matrix of T, the specific projection formula is shown below. The feature points of ith frame are first transformed into the world coordinate system by the pose matrix of the ith frame, and then the estimated value of the reprojection of these feature points under the jth frame is obtained by the pose matrix of the jth frame, where $\frac{1}{\lambda }$ denotes the depth information. The ${\left[\begin{array}{cc} u_{c_{j}} & v_{c_{j}} \end{array}\right]}^{T}$ represents the measurement value of the feature point in the jth frame in the camera coordinate system.

#### 3.1.3. Pre-integration of IMU

In the VIO algorithm, since the output frequencies of the camera and IMU are different, the vision is generally about 30 Hz, and the IMU is generally about 200 Hz. To match each other between image and IMU measurements, we need to preintegrate the information of IMU. The IMU pre-integration changes the reference coordinate system to the body coordinate system of the previous frame rather than the world frame. This information is regarded as the motion constraint provided by the IMU.

Through the pre-integration [35,36], the following motion constraints can be constructed:

(7) $$r_{IMU}=\left[\begin{array}{c} r_{p}\\ r_{q}\\ r_{v}\\ r_{b_{a}}\\ r_{b_{g}} \end{array}\right]=\left[\begin{array}{c} q_{b_{i}w}\left(p_{wb_{j}}-p_{wb_{i}}-v_{i}^{w}\Delta t+\frac{1}{2}g^{w}\Delta t^{2}\right)-\alpha_{b_{i}b_{j}}\\ 2{\left[q_{b_{j}b_{i}}⊗q_{b_{i}w}⊗q_{wb_{j}}\right]}_{im}\\ q_{b_{i}w}\left(v_{j}^{w}-v_{i}^{w}+g^{w}\Delta t\right)-\beta_{b_{i}b_{j}}\\ b_{j}^{a}-b_{i}^{a}\\ b_{j}^{g}-b_{i}^{g} \end{array}\right]$$

$\alpha_{b_{i}b_{j}},\;\beta_{b_{i}b_{j}},\;q_{b_{i}b_{j}}$ represents the pre-integration measurement and ${\left[q\right]}_{im}$ represents the imaginary part of a quaternion q. Through this step, we obtain a constraint on the IMU pre-integration information to constrain the state between two moments. For example, $p_{wb_{j}}$ and $p_{wb_{i}}$ denote the position of moment  ith and moment jth respectively. The position state is one of the system states.

#### 3.1.4. Nolinearity Optimization

When the respective cost functions are constructed, we use the nonlinear optimization algorithm to jointly optimize the objective function (14). This objective function contains three residual terms, namely, the prior constraint with marginalization information, the IMU pre-integration measurement constraint, and the visual reprojection constraint.

### 3.2. Mobile Platform Setup

Figure 2 shows the mobile platform used in the experiment. It is equipped with five modules.

### 3.3. Sensor Setup

The MEMS-based IMU is becoming more and more precise, reliable, and rugged, indicating a great future potential as the MEMS technology continues to be developed. In addition, it has a smaller size, weight, lower cost, and power and is an ideal choice for UAVs, unmanned vehicles, wearable devices, and many other applications. Considering that most fusion scenarios require lightweight hardware systems, the MEMS IMU has gradually become the mainstream. The six different IMUs we selected in the paper are all based on MEMS.

In terms of performance and usage scenarios, IMU is divided into four categories [29,33]. The first is the navigation grade, which is mainly used in spacecraft, aircraft, ships, missiles, and other rugged demand occasions. The second is the tactical grade, which is mainly used for UAV navigation and localization, smart munitions, etc. It is the most diverse and has smaller footprints and lower cost than the navigation grade. The third is the industrial grade, mainly used in industrial equipment, industrial robots, and other fields. The last is consumer-grade. IMU of this grade is a common occurrence, which is mainly used in mobile phones, wearable devices, motion-sensing games, and so on.

**Figure 3:**
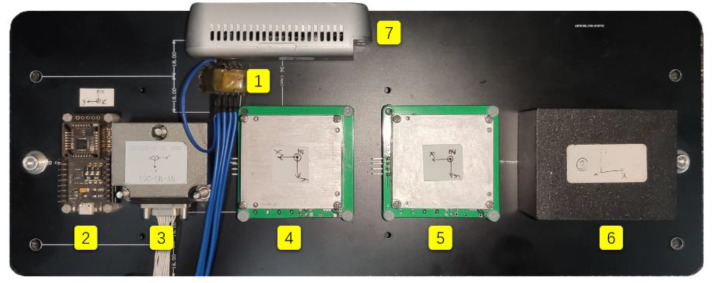
Multi-IMUs camera setup.

In the experiment, we developed six IMUs with different performances, and all were rigidly mounted on the circuit board as shown in Figure 3. Two of them are classified into consumer-grade IMU (➀ MPU6050, ➁ HI219) and four of them are classified into tactical grade IMU (➂ NV-MG-201, ➃ ADIS16488, ➄ ADIS16490, ➅ MSCRG). The module of MPU6050 is very prevailing and easy to access in the community. The nominal performance is the worst, and the price is only $1. The module of HI219, which costs $20, has been processed by the manufacturer. Many internal specifications and parameters are unknown because of the internal processing. NV-MG-201 is a tactical IMU and costs $500. The next is the two tactical products of the ADI manufacturer. The accuracy of ADIS16488 is slightly lower than ADIS16490. ADIS16488 costs $2500 and ADIS16490 costs $3000. The last IMU module named MSCRG is composed of a gyroscope from Japan and an accelerometer from Switzerland. MSCRG IMU offers high immunity to vibration and shock because of the unique resonating cos2θ ring structure for the gyroscope and is the best in class capacitive bulk MEMS accelerometer, and costs $3500. Table 2 shows the nominal specification parameters provided by the manufacturer within the six IMUs. Apart from these IMU modules, the binocular camera with the type of ➆ RealSense D435i was used to obtain the image data.

### 3.4. Sensors Parameter Configuration

#### 3.4.1. Calibration of MEMS-IMUs

**Figure 4:**
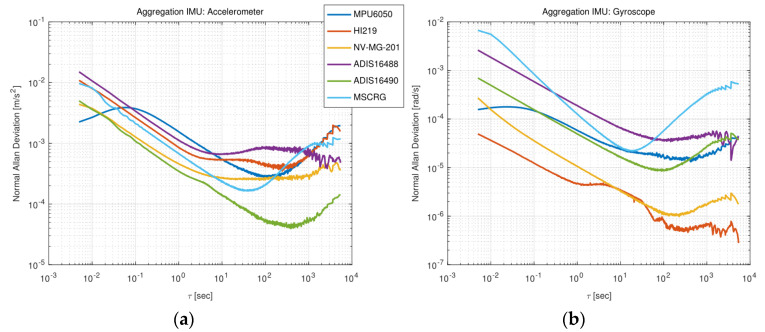
Multi IMU Allan analysis: (**a**) Description of Allan curve of six different accelerometers; (**b**) Description of Allan curve of six different gyroscopes.

Allan variance is widely applied to evaluate the noise parameters of IMU [38,39]. We used the open-sourced tool kalibr_allan [40] to analyze Allan’s deviation of each IMU. The Allan curve was plotted in Figure 4, and the Allan result was summarized in Table 3. If bias stability is taken as the evaluation standard, their accelerometer performance ranking from low to high is roughly: ADIS16488, HI219, MPU6050, NV-MG-201, MSCRG, ADIS16490. For gyroscopes, the ranking is ADIS488, MSCRG, MPU6050, ADIS16490, NV-MG-201, and HI219. In addition, there is no strong correlation with price. Although there is no strict standard, the tactical IMU has lower bias stability. Surprisingly, as a consumer-grade HI219 gyroscope, it has the lowest bias stability.

In-run bias stability, often called the bias instability, is an indication of how the bias will drift during a period of time at a certain temperature and is a considerable characterization parameter. For bias repeatability, it represents the dispersion of the sensor’s bias at each powerup. How similar is the bias at each powerup of IMU? Because the thermal, physical, electrical, etc., will not be exactly the same during each powerup, there will be fluctuations in the bias. If the bias repeatability is greater, the bias consistency is worse and will affect the accuracy of the system. The inertial navigation system can estimate the bias after each powerup. The noise represents the measurement noise of the sensor. Wiener process is usually used to model the process of bias changing continuously with time, which is called bias random walk. The noise and bias random walk constitute the diagonal covariance matrix of the sensor noise term.

Due to the incompleteness of the IMU measurement model, the parameters above cannot be directly used in the configuration parameters of VINS-Fusion after discretization, otherwise, the trajectory will drift. We appropriately enlarge and adjust the discretized parameters to obtain their configuration parameters. To control the variables in the experiment and consider only the different performances of IMUs, we average the configuration parameters and obtained a common configuration parameter. In this way, only the impact of IMU on the fusion algorithm is considered. The common configuration parameters are acc_ n(0.170)*,* gyr_ n(0.014)*,* acc_ w(0.008)*,* gyr_ w(0.00042).

#### 3.4.2. Camera-IMU Temporal-Spatial Calibration

The off-line calibration of cameras and IMU is a widely studied problem. In order to effectively fuse visual information with IMU information, we need to unify the camera and IMU into a certain coordinate system, that is, the spatial calibration of the camera and IMU. Spatial calibration bridges the gap between the data in different coordinate systems. In addition, since the camera and IMU are triggered separately under different clock sources, and there are problems such as transmission delay and CPU overload, it is necessary to correct the time offset between the camera and IMU. We exploited the open-sourced calibration tool Kalibr [41,42] to obtain the spatial transformation. As to the time offset error between IMU and cameras, we enabled the VINS_ Fusion’s online calibration algorithm [43].

#### 3.4.3. Sensors Frequency

For the sake of experimental consistency, the output frequency of the six IMUs was uniformly set to 200 Hz and the frequency of the camera was 30 Hz. In addition, the resolution of the left and right images was 640×480.

#### 3.4.4. Loop Closure

In the experiment, the goal was to explore the fusion of vision and inertial independently. In consideration of the visual loop would calibrate the accumulated error, loop-closure detection was not enabled here.

### 3.5. Evaluation Scenario

#### 3.5.1. Weak Texture in Corridor

**Figure 5:**
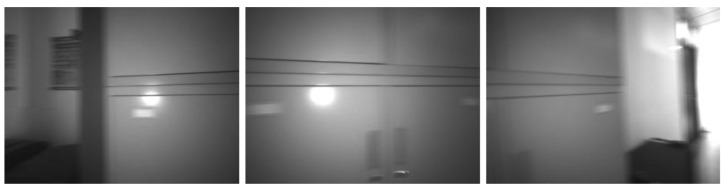
Weak texture in corridor.

**Figure 6:**
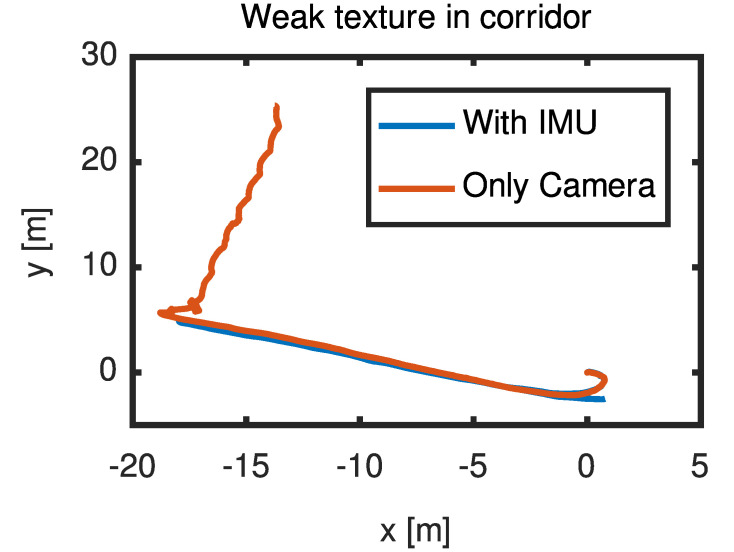
The trajectory with and without IMU auxiliary in weak texture.

We chose the corridor as the test scenario, walked straight for a distance, and returned the same way. There was a weak texture area as shown in Figure 5 at the corner, which lasted for 0.5 s. From Figure 6, it can be found that the IMU’s assistance makes the track coincide, and only the camera will directly give the wrong track.

#### 3.5.2. Uniform Velocity Motion State

**Figure 7:**
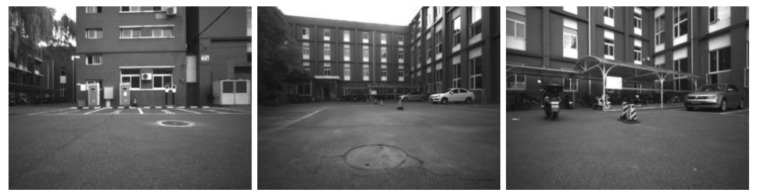
The outdoor environment without strong sunlight.

**Figure 8:**
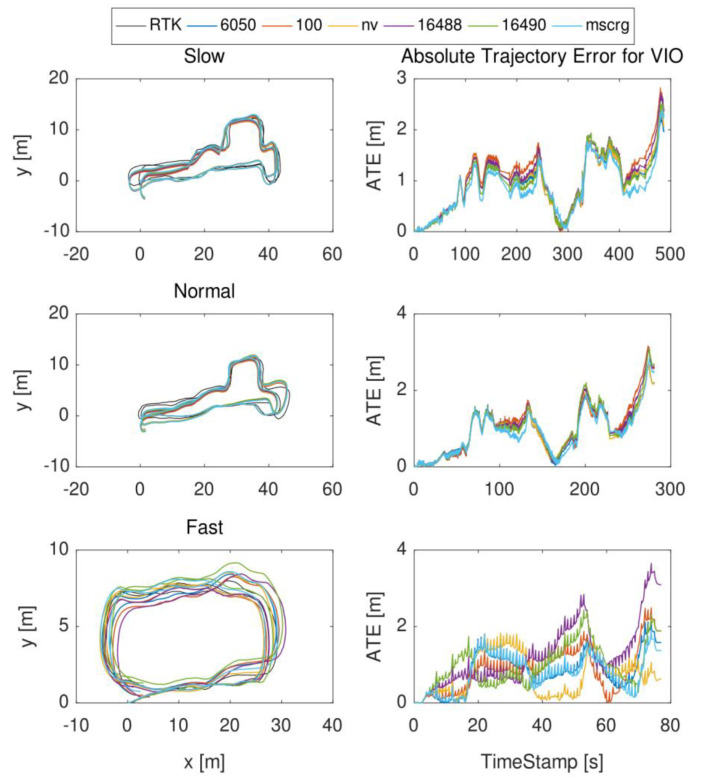
The trajectory of uniform velocity motion state (slow, normal, fast): 6050, 100, nv, 16488, 16490, and mscrg represent MPU6050, HI219, NV−MG−201, ADIS16488, ADIS16490, and MSCRG, respectively. The left represents the ground truth trajectory and the output trajectory of visual−inertial odometry which is based on six different IMU (The *x*−axis and *y*−axis represent the 2D plane in the experimental environment). The right represents the ATE results for VIO. (The *x*−axis and *y*−axis represent the running timestamp and error, respectively.) (Other similar result graphs also follow this rule).

In these experiments, due to the high-precision RTK ground truth can be provided outdoors, we selected the environment as shown in Figure 7 for evaluation. There were three uniform velocity motion modes: slow, normal, and fast, and they moved for 300, 200, and 120 s, respectively. To maintain experimental consistency, each motion state was evaluated five times. One of the trajectory diagrams and ATE diagrams are drawn as shown in Figure 8.

#### 3.5.3. Alternating Acceleration and Deceleration Motion State

**Figure 9:**
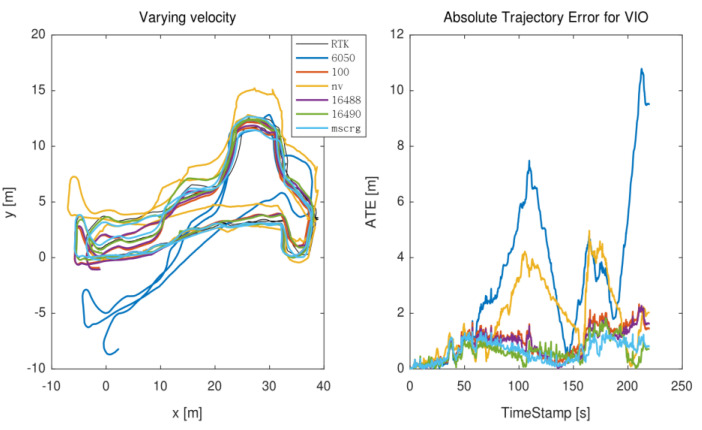
The trajectory of varying velocity motion state.

In this scenario, the wheeled robot ceaselessly kept accelerating and decelerating in order to motivate IMU. Figure 9 plots the trajectory and the ATE error. Similarly, to maintain experimental consistency, this motion state was evaluated five times. It can be clearly reported that MPU6050 has a poor performance and larger drift.

#### 3.5.4. Spin Move Forward Motion State

**Figure 10:**
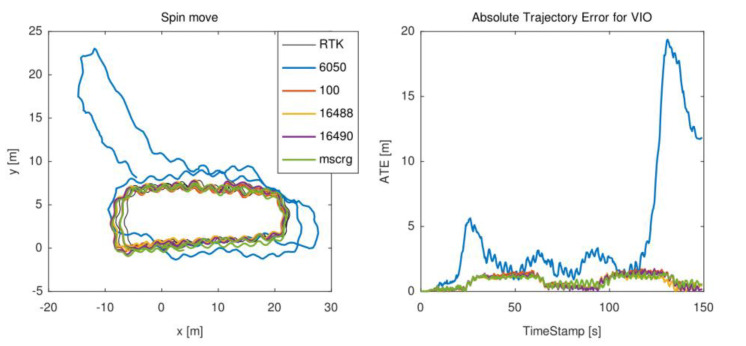
The trajectory of the spin move forward motion state. The waveform trajectories can reflect this motion state.

In this case, the wheeled robot moved forward with frequent rotation in order to motivate IMU. The trajectory shown in Figure 10 can reflect this movement. This motion was evaluated eleven times due to the complexity of the motion state. In this case, MPU6050 is easy to crash due to rapid rotation.

#### 3.5.5. Strong Sun Light Scene

**Figure 11:**
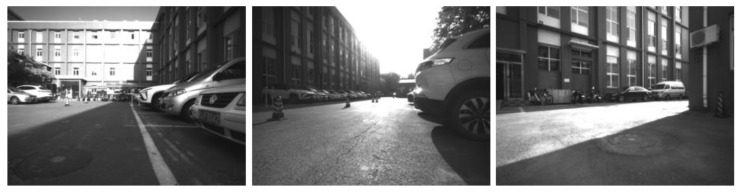
The strong sunlight scene in the experiment.

**Figure 12:**
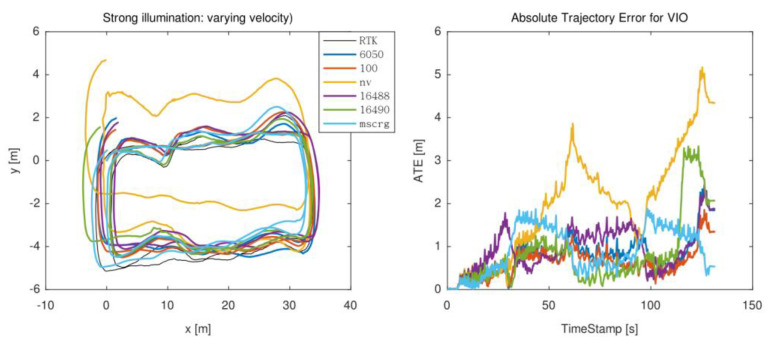
The trajectory of a strong sunlight scene.

In this scene, the situation in a strong illumination environment was evaluated. As shown in Figure 11, there was obviously strong light in the environment. A cross-over study to evaluate the performance of different motion states under strong light was conducted. The trajectory under the variable speed scene is chosen as shown in Figure 12.

#### 3.5.6. Long Term Scene

**Figure 13:**
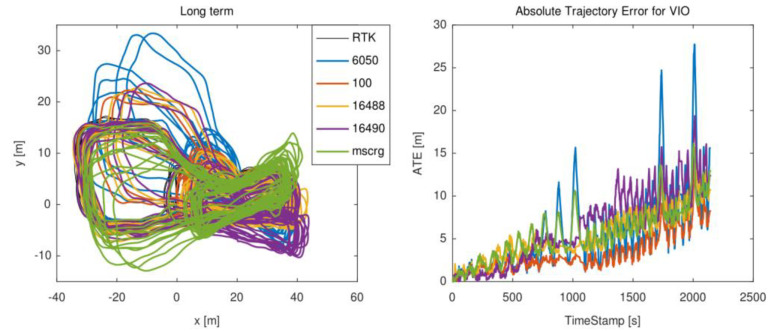
The trajectory of the long−term scene.

In long-term test experiment, which included three forms of motion: constant speed, variable speed, and spin move, was performed for 30 min. The trajectory is shown in Figure 13. From Table 4, the HI219 IMU improved the localization accuracy of the fusion system, while other IMUs deteriorated the accuracy of the system.

## 4. Results and Analysis

Through the preceding experiments and according to Table A1, Table A2, Table A3, Table A4, Table A5 and Table A6 in Section of Appendix A, we summarize the votes of accuracy improvement of each IMU as shown in Table 5. If an IMU improved the accuracy the most in an evaluation scenario, we would vote for this IMU and count the votes. For example, among the fifteen evaluations in uniform velocity scenes, the ADIS16488 performed best only once in the six IMUs. As for ADIS16490, the best performance was three times.

Some results are revealed based on the evaluation: (1) In the weak texture scenario over short time intervals, the localization posture is significantly improved with the aid of IMU, and only leveraging the camera will be defeated because of the absence of visual constraints. (2) The IMU’s incentive is relatively small in the nearly constant speed scenario, there is no salient difference between IMUs with different performances, consumer IMUs will also perform well, and better IMUs do not show more visible advantages. (3) In the case of varying speed scenarios, the assistance of an accelerometer is needed. The IMU with the excellent specification of bias stability has better performance, such as ADIS16490 and MSCRG. (4) In the spin move situation, the gyroscope is needed. At this time, the HI219 and MSCRG have better results. (5) These performance trends are maintained in the cross experiments facing a strong sunlight environment. (6) In the 30 min long-term test, only the HI219 improves the odometry accuracy of the fusion system. Among these results, it is surprising that HI219, as a consumer-grade also has a good performance.

### 4.1. Turntable Test

**Figure 14:**
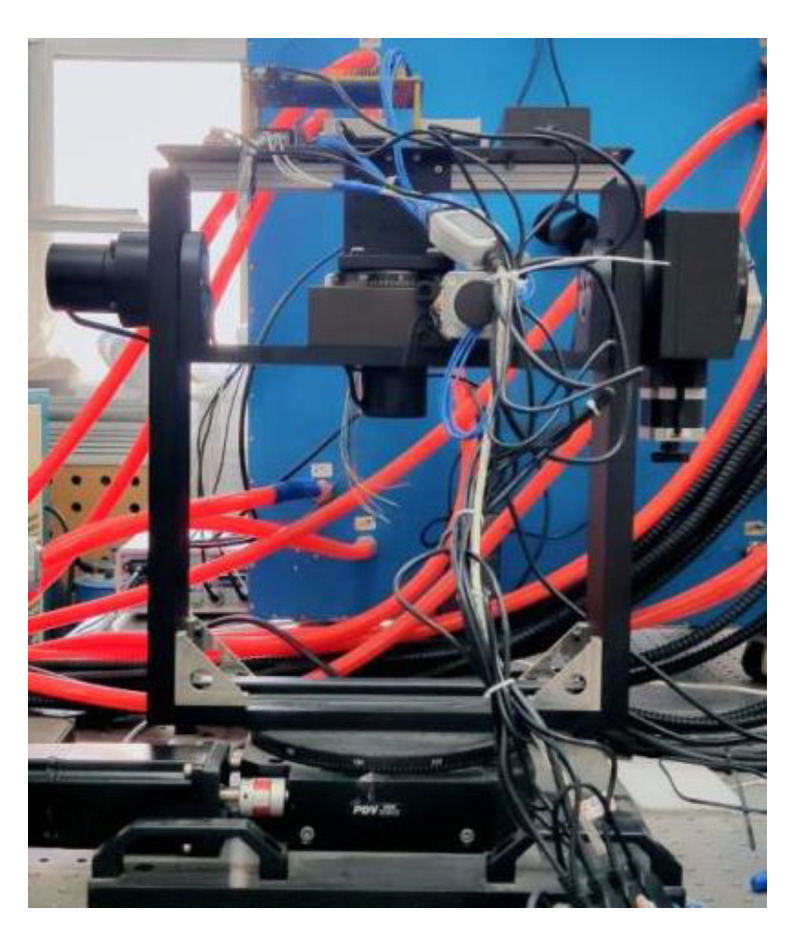
The multiaxial turntable with multi-IMUs camera platform is rigidly mounted.

**Figure 15:**
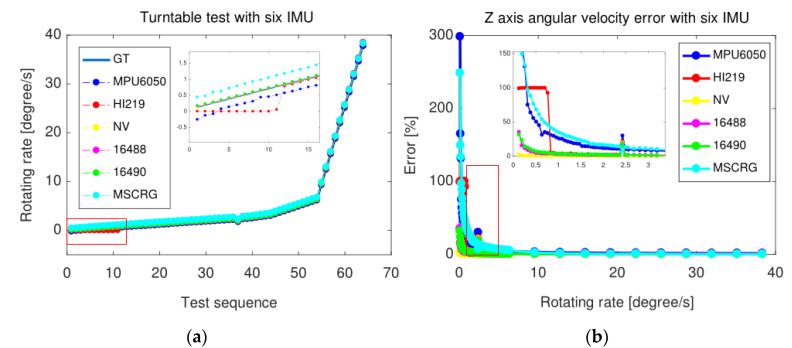
The results of multiple IMUs on turntable: (**a**) Description of six MEMS-IMUs angular velocity test results with a multiaxial turntable; (**b**) Description of Z-axis angular velocity absolute error of six MEMS-IMUs.

In order to make the results clearer, the professional multiaxial turntable as shown in Figure 14 was used to uniformly measure the angular velocity of the six IMUs. As can be seen from Figure 15a, the output of HI219 is always maintained at zero when the angular velocity is less than 0.83°/s, there is a threshold to perceive the rotational motion, while other sensors output their perceived angular velocity although the value is amiss. For example, when the robot is stationary, orientation will not drift due to the zero-bias of the gyroscope. This phenomenon makes HI219 bring less drift and results in the lowest bias stability of the gyroscope as shown in Figure 4b. In addition, when the angular velocity is greater than 16°/s, except mpu6050, the rest IMUs’ error has been reduced to less than 9%, and their error discrimination is very small as shown in Figure 15b, resulting in the algorithm error between them not being very remarkable.

It should be noted that the triaxial gyroscope sensor is actually composed of triaxial identical gyroscopes placed in orthogonal directions, hence the rotation of the gyroscope around the *Z*-axis was measured, and there are more cases around the gravity direction in-plane motion.

### 4.2. Quantitative Analysis

The measurement model of the accelerometer and gyroscope is given follows:

(8) $${\tilde{w}}^{b}=w^{b}+b^{g}+n^{g}$$

(9) $${\tilde{a}}^{b}=q_{bw}\left(a^{w}+g^{w}\right)+b^{a}+n^{a}$$

${\tilde{w}}^{b}$ and ${\tilde{a}}^{b}$ represent the measurements of the gyroscope and accelerometer, respectively. $w^{b}$ and $a^{w}$ represent the ideal value of the gyroscope and accelerometer, respectively. Due to various factors, measurements are affected by gyroscope bias $b^{g}$, acceleration bias $b^{a}$, and noise n. In addition, we assume that the noise in gyroscope and acceleration measurements are Gaussian, that is, $n^{g}\sim N\left(0,\;\sigma_{g}^{2}\right)$, $n^{a}\sim N\left(0,\;\sigma_{a}^{2}\right)$. As for the bias, gyroscope bias and acceleration bias are modeled as a random walk, whose derivatives are gaussian, $n_{b_{g}}\sim N\left(0,\sigma_{b_{g}}^{2}\;\right)$, $n_{b_{a}}\sim N\left(0,\sigma_{b_{a}}^{2}\;\right)$, so we can obtain:

(10) $${\dot{b}}^{g}=n_{b_{g}}$$

(11) $${\dot{b}}^{g}=n_{b_{g}}$$

We can obtain the above four noise coefficients ($n^{g}$, $n^{a}$, $n_{b_{g}}$, $n_{b_{a}}$) through the Allan analysis in Section 3.4.1. These configuration parameters constitute the noise covariance matrix Q:

(12) $$Q=\left[\begin{array}{cc} \begin{array}{ccc} \sigma_{a}^{2} & 0 & 0\\ 0 & \sigma_{g}^{2} & 0\\ 0 & 0 & \sigma_{a}^{2} \end{array} & \begin{array}{ccc} 0 & 0 & 0\\ 0 & 0 & 0\\ 0 & 0 & 0 \end{array}\\ \begin{array}{ccc} 0 & 0 & 0\\ 0 & 0 & 0\\ 0 & 0 & 0 \end{array} & \begin{array}{ccc} \sigma_{g}^{2} & 0 & 0\\ 0 & \sigma_{b_{a}}^{2} & 0\\ 0 & 0 & \sigma_{b_{g}}^{2} \end{array} \end{array}\right]$$

We can update the covariance matrix of pre-integration by the transition matrix F, V, and noise matrix  Q, see Appendix B for specific elements of F and V:

(13) $$P_{k+1}=FP_{k}F^{T}+VQV^{T}$$

Similarly, there is a covariance matrix for visual information. These covariance matrices represent the noise of IMU information and visual information. We can optimize the system state X  through the following cost function:

(14) $$r_{obj}=min\left\{\|r_{p}-J_{p}{X\|}^{2}+\|r_{IMU}{\left(Z^{IMU},X\right)\|}_{p_{IMU}}^{2}+\|r_{visual}{\left(Z^{visual},X\right)\|}_{P_{visual}}^{2}\right\}$$

It should be noted that through the covariance matrix $P_{IMU}$ and $P_{visual}$, the Euclidean distance is converted into Mahalanobis distance. It fixes the problem of inconsistent and related dimensions in European distance. Although the information is very different between the IMU and visual, they can be optimized in the same cost function (14) through Mahalanobis distance. In this way, the residual information of IMU and visual is statistically equivalent to each other. For visual information, it is consistent throughout the evaluation experiment, so only IMU information affects the accuracy of visual-inertial odometry.

Through the algorithm evaluation results in Table A1, Table A2, Table A3, Table A4, Table A5 and Table A6 in the section of Appendix A, we summarized the accuracy of the localization improved by adding IMU and obtained the improvement under the following four main motion scenes just as shown in Table 6.

Combined with quantitative results, in the uniform velocity situation, the lifting amplitude of each IMU is in the range of 0.1 m. In the varying velocity situation, the IMU produced by ADI has increased the most, which is better than MPU6050 in this scenario. In spin move situations, because of the special processing of HI219 and the superiority of the MSCRG structure, the accuracy is greatly improved by their assistance. In the strong illumination situation, there is little difference between them. Except for NV-MG-201, the lifting effect of different IMUs is 0.15m. The most expensive IMU is 2.5 cm higher in accuracy than the cheapest one.

In summary, the improvement of fusion accuracy with different grade MEMS-IMU is limited in these experimental scenarios, the consumer-grade IMU can also have an excellent result. The improvement of accuracy depends more on the algorithm.

As shown in formula (14), if the measurement noise of IMU is smaller, the theoretical accuracy will be higher. However, when the difference in measurements between these IMU is small, the difference in residual information between them is smaller. Due to the weighting effect in formula (14), this results in limited differences in accuracy.

## 5. Conclusions

In general, there are many internal and external factors that affect the IMU. We can obtain the representative parameters through the Allan variance method to quantitatively analyze the performance of IMU. However, the method cannot represent all performance in actual applications. In this paper, many scenario experiments were conducted, and the professional turntable was employed to analyze the error of six IMUs. The following conclusions are reached:

The assistance of IMU can improve the accuracy of multi-sensor fusion, and is more notable in weak texture scenes. In the constant speed scene, there is no obvious difference between IMUs with different performances. Under the excitation of rotation, acceleration, and deceleration, IMUs with excellent performance will have higher accuracy and are more stable. Owing to the lower bias stability and noise, making their performance more robust. The improvement of fusion accuracy is not directly proportional to the price with regard to the expensive ADIS16490 IMU, even so, it is more versatile in various scenarios. For HI219, a consumer IMU, there is a threshold for sensing rotation motion, performing well in rotation scenes, which may provide a reference for the processing of the algorithm. At the same time, according to the MSCRG IMU results, IMU with resistance to vibration and impact is more needed in the situation of frequently strenuous movement.

## Figures and Tables

**Table 1:** Scenario sequences.

Normal Illumination						Strong Illumination	Corridor
uniform_velocity (approximate)			varying_velocity	spin_move	long_term	slow, normal, fast, varying_velocity, spin_move	weak_ texture
slow	normal	fast	alternating acceleration and deceleration	spin_move _forward	30 min		

**Table 2:** Nominal specifications of MEMS-IMUs in the experiment.

Grade	IMU Type		Range	Bandwidth	Bias Stability	Bias Repeatability	Non linearity	Resolution	Price $
Consumer	MPU6050	Acc	±4 g	\	\	\	0.5%	0.06 mg/LSB	1
		Gyro	±2000°/s	\	\	\	0.2%	0.061°/s/LSB	
Consumer	HI219	Acc	±16 g	\	\	\	\	\	20
		Gyro	±2000°/s	\	\	\	\	\	
Tactical	NV-MG-201	Acc	±30 g	100 Hz	80 μg	100 μg	0.03%	\	500
		Gyro	±500°/s	80 Hz	0.8°/h	0.8°/h	0.03%	\	
Tactical	ADIS16488	Acc	±18 g	330 Hz	0.1 mg	±16 mg	0.5%	0.8 mg/LSB	2500
		Gyro	±450°/s	330 Hz	6.25°/h	±0.2°/s	0.01%	0.02°/s/LSB	
Tactical	MSCRG	Acc	±30 g	200 Hz	45 μg	3.6 mg	0.3%	0.0572 mg/LSB	3000
		Gyro	±300°/s	75 Hz	\	0.07°/s	0.15%	0.03125°/s/LSB	
Tactical	ADIS16490	Acc	±8 g	750 Hz	3.6 μg	±3.5 mg	1.6%	0.5 mg/LSB	3500
		Gyro	±100°/s	480 Hz	1.8°/h	0.05°/s	0.3%	0.005°/s/LSB	

**Table 3:** Experimental calibration results of MEMS-IMUs.

			Accelerometer		Gyroscope	
IMU Type	Noise	Bias Stability	Bias Random Walk	Noise	Bias Stability	Bias Random Walk
MPU6050	0.000995	0.00035	0.000053	0.000048	0.000012	0.000001
HI219	0.001420	0.00040	0.000043	0.000005	5.00 × 10^-7^	0.000001
NV-MG-201	0.000508	0.00028	0.000028	0.000014	7.00 × 10^-7^	0.000001
ADIS16488	0.002999	0.00060	0.000014	0.000252	0.000034	0.000001
ADIS16490	0.000378	0.000034	0.000005	0.000051	8.00 × 10^-6^	0.000001
MSCRG	0.000701	0.00015	0.000040	0.000205	1.80 × 10^-5^	0.000013

**Table 4:** The ATE results for the long-term scene with six different MEMS-IMUs.

Long Term		MPU6050	HI219	NV-MG-201	ADIS16488	ADIS16490	MSCRG
Long_term	Mean	5.72/3.9934	3.5327/4.0316↑	Fail	5.5085/4.0496	6.4623/4.0012	5.7496/3.9860
	Median	4.5199/2.7960	2.6263/2.8203↑	Fail	4.9339/2.8902	7.1918/2.8369	5.6274/2.8210
	RMSE	7.4087/5.5448	4.2584/5.6121↑	Fail	6.3012/5.5689	7.8725/5.5018	6.5565/5.4862

**Table 5:** The votes for all scenarios with six different MEMS-IMUs.

Scenarios	Number of Experiments	MPU6050	HI219	NV-MG-201	ADIS16488	ADIS16490	MSCRG
Uniform_velocity (approximate)	15	3	0	4	1	3	0
Varying_velocity	5	0	0	0	1	2	1
Spin_move	11	0	3	0	2	1	3
Strong_illumination	7	1	3	0	2	1	0
Long_term	1	0	1	0	0	0	0

**Table 6:** Improvement of average localization accuracy after adding IMU in different scenarios. Unit: m (we considered the situation of improvement of accuracy. Each item represents accuracy improvement in the situation of IMU auxiliary relative to the situation of only visual localization. For example, the auxiliary of MPU6050 improves the accuracy in uniform scenes by 0.1031 m).

Scenes	MPU6050	HI219	NV-MG-201	ADIS16488	ADIS16490	MSCRG
Uniform_velocity	0.1031	0.1019	0.1692	0.0927	0.0783	0.0955
Varying_velocity	0.0432	0.0000	0.0000	0.1874	0.1952	0.1477
Spin_move	0.1895	0.7093	0.0000	0.1874	0.1762	0.8175
Strong_illumination	0.1368	0.1769	0.0386	0.1634	0.1619	0.1381

## Appendix A

This appendix records the absolute trajectory error (ATE) results evaluated under each scenario. Each column represents different IMUs, and each row represents the algorithm accuracy including mean, median, and RMSE about ATE. Each cell represents the error results with IMU assistance and without IMU assistance (**0.9124**/1.1250 ↑). The bold represents that the accuracy has been improved with the aid of IMU. The symbol ↑ represents the IMU with the greatest accuracy improvement in this evaluation. We vote for the IMU through the symbol and make statistics according to the number of votes.

### Appendix A.1. Uniform Scene

**Table A1:** The algorithm errors for slow velocity scene with six different MEMS-IMUs. (Bold indicates that the accuracy was improved compared with using only visual information.)

Slow	ATE	MPU6050	HI219	NV-MG-201	ADIS16488	ADIS16490	MSCRG
Slow0	Mean	**0.9124**/1.1250 ↑	**1.0151**/1.1772	**0.9344**/1.1129	**0.9871**/1.0713	**0.9177**/1.0534	**0.8240**/1.0076
	Median	**1.0192**/1.2815 ↑	**1.1606**/1.3546	**1.0385**/1.2773	**1.0562**/1.2002	**0.9499**/1.1479	**0.7879**/1.0531
	RMSE	**1.0501**/1.3076 ↑	**1.181**/1.3814	**1.0769**/1.2943	**1.1458**/1.2414	**1.0694**/1.2269	**0.9571**/1.1748
Slow1	Mean	0.9982/0.9895	**0.9016**/1.0130	2.4118/0.9948	**0.9532**/0.9619	**0.7919**/0.9347 ↑	0.9319/0.8955
	Median	1.1267/1.1155	**0.9185**/1.1656	2.0879/1.1279	**1.0373**/1.0720	**0.7604**/1.0316 ↑	1.0083/0.9740
	RMSE	1.196/1.1951	**1.0977**/1.2237	3.0902/1.2027	**1.1436**/1.1628	**0.9512**/1.1286 ↑	1.1177/1.0811
Slow2	Mean	**0.8791**/0.9547	1.082/0.9788	**0.7747**/0.9659 ↑	0.9614/0.9266	0.8861/0.8967	0.8562/0.8535
	Median	**0.9678**/1.0318	1.1646/1.0415	**0.8479**/1.0376 ↑	0.9768/1.0005	0.9715/0.9713	0.901/0.9153
	RMSE	**1.0553**/1.1400	1.3208/1.1690	**0.9664**/1.1542 ↑	1.1714/1.1048	1.0685/1.0664	1.0334/1.0132
Slow3	Mean	**1.0825**/1.1577 ↑	1.1673/1.1765	1.1591/1.1613	1.1518/1.1288	1.1141/1.1078	**1.0253**/1.0660
	Median	**1.0354**/1.0487 ↑	1.1027/1.0637	1.0527/1.0424	1.0396/1.0171	1.0445/1.0383	**1.0080**/0.9922
	RMSE	**1.3088**/1.4197 ↑	1.4243/1.4455	1.442/1.4278	1.3992/1.3878	1.3479/1.3576	**1.2503**/1.3082
Slow4	Mean	1.0988/1.0994	1.1458/1.1457	1.1683/1.1205	1.1014/1.0757	1.0066/1.0275	0.9774/0.9730
	Median	1.2009/1.2267	1.2493/1.2761	1.3026/1.2539	1.1807/1.1815	1.1067/1.1086	1.0918/1.0319
	RMSE	1.2709/1.2743	1.331/1.3305	1.3542/1.3006	1.2814/1.2486	1.1631/1.1923	1.1341/1.1300

**Table A2:** The algorithm errors for normal velocity scene with six different MEMS-IMUs.

Normal	ATE	MPU6050	HI219	NV-MG-201	ADIS16488	ADIS16490	MSCRG
Normal0	Mean	**0.9012**/0.9403	0.9512/0.9569	**0.8939**/0.9518 ↑	0.9365/0.9214	0.9244/0.8931	**0.8449**/0.8592
	Median	**1.0108**/1.0967	1.0405/1.1298	**0.9690**/1.1193 ↑	0.9933/1.0597	0.9363/0.9941	**0.8833**/0.9112
	RMSE	**1.0628**/1.1203	1.1431/1.1423	**1.0515**/1.1363 ↑	1.1215/1.0987	1.1086/1.0650	**1.0133**/1.0267
Normal1	Mean	1.008/0.9150	1.0002/0.9375	1.3758/0.9261	1.1672/0.8910	1.0215/0.8685	0.8925/0.8311
	Median	1.0307/0.9896	0.9848/1.0287	1.2971/0.9995	1.1925/0.9519	1.0452/0.9204	0.8556/0.8462
	RMSE	1.1937/1.0577	1.1769/1.0795	1.6474/1.0696	1.383/1.0324	1.2145/1.0143	1.0726/0.9770
Normal2	Mean	**1.1009**/1.1163	**1.1184**/1.1337	1.8117/1.1198	**1.0196**/1.0886 ↑	1.1002/1.0666	1.0511/1.0275
	Median	**1.2592**/1.3108	**1.2650**/1.3211	1.7949/1.3030	**1.0272**/1.2722 ↑	1.2695/1.2555	1.2361/1.2060
	RMSE	**1.2867**/1.3038	**1.3064**/1.3262	2.1023/1.3094	**1.1777**/1.2700 ↑	1.2763/1.2417	1.2189/1.1940
Normal3	Mean	1.094/1.0652	1.2055/1.0787	2.2835/1.0662	1.0922/1.0416	1.1508/1.0278	1.0364/1.0003
	Median	1.1548/1.0851	1.2595/1.0977	1.5083/1.0815	1.0228/1.0473	1.1174/1.0257	0.9945/0.9829
	RMSE	1.2522/1.2151	1.3812/1.2291	3.0337/1.2164	1.2712/1.1904	1.3275/1.1778	1.1962/1.1522
Normal4	Mean	**1.0675**/1.1392	**1.0915**/1.1223	**0.8973**/1.1497 ↑	**1.078**/1.1115	**1.0762**/1.0801	**0.9196**/1.0357
	Median	**1.1940**/1.1446	**1.2174**/1.1190	**1.0327**/1.1515 ↑	**1.1788**/1.1033	**1.1431**/1.3724	**0.9527**/1.0138
	RMSE	**1.2755**/1.3698	**1.3080**/1.5011	**1.0475**/1.3830 ↑	**1.3186**/1.3383	**1.3034**/1.3004	**1.0934**/1.2487

**Table A3:** The algorithm errors for fast velocity scene with six different MEMS-IMUs.

Fast	ATE	MPU6050	HI219	NV-MG-201	ADIS16488	ADIS16490	MSCRG
Fast0	Mean	**0.9561**/1.0820	**0.9762**/1.1100	4.9392/1.0902	**0.8968**/1.0514	**0.8359**/1.0234 ↑	**0.8661**/0.9850
	Median	**0.9923**/1.1120	**1.0133**/1.1815	4.8637/1.1358	**0.8401**/1.0587	**0.8179**/1.0049 ↑	**0.8228**/0.9544
	RMSE	**1.1026**/1.2503	**1.1461**/1.2789	6.156/1.2576	**1.0471**/1.2152	**0.9937**/1.1875 ↑	**1.0135**/1.1462
Fast1	Mean	1.6826/1.1371	1.5799/1.1523	5.346/1.1433	1.1282/1.1127	1.1426/1.0887	1.3559/1.0530
	Median	1.8629/1.2633	1.6132/1.2948	5.7653/1.2849	1.1536/1.2200	1.0826/1.1825	1.5837/1.1135
	RMSE	1.974/1.3432	1.8504/1.3582	6.4123/1.3509	1.3272/1.3150	1.3627/1.2873	1.5819/1.2470
Fast2	Mean	1.1355/1.0110	1.2949/1.0426	3.7447/1.0228	0.9974/0.9864	**0.9028**/0.9466 ↑	1.0003/0.9087
	Median	1.2935/1.0741	1.3555/1.1110	4.0317/1.0989	1.0213/1.0229	**0.8274**/0.9488 ↑	1.0575/0.8987
	RMSE	1.349/1.1764	1.5494/1.2102	4.5396/1.1893	1.1687/1.1504	**1.0330**/1.1087 ↑	1.1613/1.0701
Fast3	Mean	**0.6902**/0.8320 ↑	**0.8223**/0.8551	1.1044/0.8403	1.078/0.8108	1.6313/0.7893	0.766/0.7615
	Median	**0.7204**/0.7722 ↑	**0.7826**/0.8011	0.8646/0.7838	0.9344/0.7538	1.513/0.7444	0.7928/0.7227
	RMSE	**0.8458**/0.9759 ↑	**0.9655**/0.9997	1.3657/0.9853	1.3591/0.9558	1.9245/0.9354	0.8762/0.9094
Fast4	Mean	**0.8369**/0.8511	0.9637/0.8636	**0.7259**/0.8507 ↑	1.2174/0.8262	0.9624/0.8154	**0.7646**/0.7957
	Median	**0.8422**/0.9188	0.8478/0.9566	**0.7117**/0.9280 ↑	1.0523/0.8803	0.8593/0.8364	**0.7640**/0.8134
	RMSE	**0.9892**/0.9900	1.0377/1.0045	**0.8949**/0.9897 ↑	1.4716/0.9606	1.1077/0.9503	**0.9042**/0.9314

### Appendix A.2. Alternating Acceleration and Deceleration

**Table A4:** The algorithm errors for varying speed scene with six different MEMS-IMUs.

Varying	ATE	MPU6050	HI219	NV-MG-201	ADIS16488	ADIS16490	MSCRG
Vary0	Mean	3.1442/0.8565	0.9675/0.8804	1.8236/0.8614	0.8628/0.8271	**0.6212**/0.7974 ↑	**0.6321**/0.7578
	Median	2.5754/0.9286	1.0943/0.9876	1.6019/0.9516	0.9879/0.8873	**0.5668**/0.8196 ↑	**0.6858**/0.7547
	RMSE	4.0895/0.9926	1.1185/1.0199	2.231/0.9974	1.0088/0.9577	**0.7329**/0.9255 ↑	**0.7222**/0.8818
Vary1	Mean	**0.7834**/0.8052	0.9755/0.8242	2.3572/0.8106	**0.7476**/0.7821	0.8797/0.7497	**0.6229**/0.7410 ↑
	Median	**0.7256**/0.7473	1.0616/0.8000	1.9911/0.7621	**0.6495**/0.7188	0.8082/0.6774	**0.5938**/0.6674 ↑
	RMSE	**0.9223**/0.9398	1.1345/0.9642	3.0779/0.9472	**0.8692**/0.9180	1.0088/0.8914	**0.7273**/0.8898 ↑
Vary2	Mean	0.9991/0.8205	1.0003/0.8398	2.2953/0.8252	0.9603/0.7957	0.981/0.7751	0.7688/0.7450
	Median	0.8234/0.8659	0.9777/0.9120	1.8541/0.8803	1.0685/0.8254	0.9861/0.7926	0.8802/0.7519
	RMSE	1.2706/0.9907	1.2199/1.0138	3.0457/0.9972	1.1727/0.9611	1.2231/0.9373	0.9312/0.9032
Vary3	Mean	0.9397/0.8479	0.9267/0.8602	1.8007/0.8501	**0.5774**/0.8259 ↑	**0.7250**/0.8224	**0.7491**/0.7976
	Median	0.9734/1.0072	1.056/1.0121	1.7407/0.9996	**0.6014**/0.9764 ↑	**0.7392**/0.9433	**0.7941**/0.8830
	RMSE	1.1398/1.0319	1.123/1.0467	2.2473/1.0348	**0.6782**/1.0042 ↑	**0.8334**/1.0028	**0.8507**/0.9718
Vary4	Mean	**0.7687**/0.8249	0.8873/0.8399	1.5669/0.8287	0.8343/0.8034	**0.6626**/0.7892 ↑	0.7768/0.7647
	Median	**0.8952**/0.8233	0.8828/0.8683	1.4704/0.8313	0.8051/0.7883	**0.6469**/0.7745 ↑	0.815/0.7513
	RMSE	**0.9163**/0.9852	1.0959/1.0021	1.919/0.9896	0.9668/0.9599	**0.7201**/0.9438 ↑	0.9554/0.9158

### Appendix A.3. Spin Move forward

**Table A5:** The algorithm errors for a spin move forward scene with six different MEMS-IMUs.

Spin_move	ATE	MPU6050	HI219	NV-MG-201	ADIS16488	ADIS16490	MSCRG
Spin_move0	Mean	9.3128/1.3158	**0.8370**/1.3188	Fail/1.3027	**0.6069**/1.2982 ↑	**0.6830**/1.2838	Fail/1.2548
	Median	7.5538/1.4859	**0.9094**/1.4970	Fail/1.4748	**0.6195**/1.4543 ↑	**0.6371**/1.4289	Fail/1.4066
	RMSE	11.8427/1.4396	**0.9383**/1.4431	Fail/1.4252	**0.6891**/1.4192 ↑	**0.7805**/1.4044	Fail/1.3735
Spin_move1	Mean	514.9947/1.1230	1.1685/1.1024	3537/1.0896	1.4894/1.1359	1.9135/1.0979	**0.9042**/1.1532 ↑
	Median	464.7805/1.0073	1.2253/1.0144	2783/1.0117	1.4236/1.0003	1.6958/0.9978	**0.7804**/1.0180 ↑
	RMSE	686.0213/1.3204	1.3792/1.2997	4827/1.2765	1.6514/1.3381	2.2048/1.2858	**1.0620**/1.3512 ↑
Spin_move2	Mean	2463/2.4171	**1.9738**/2.3948 ↑	3853/2.4889	5.6023/2.3980	10.8429/2.4722	98.577/2.3734
	Median	1842/2.4354	**2.3318**/2.3874 ↑	3004/2.4396	5.8112/2.3848	11.944/2.3987	144.8365/2.4576
	RMSE	3502/2.8896	**2.3169**/2.8421 ↑	5261/3.0017	6.1396/2.8688	12.4/3.0149	117.7245/2.8578
Spin_move3	Mean	762.9145/4.8219	5.7136/4.9950	1831/4.8104	211.801/3.9853	187.2453/4.6046	**4.0960**/4.7584 ↑
	Median	1024/4.6508	6.3944/4.9114	1795/4.6099	296.713/4.1932	254.9551/4.3790	**4.3375**/4.7746 ↑
	RMSE	938.5596/5.3919	6.1691/5.5646	2412/5.3985	247.016/4.1072	217.4266/5.1701	**4.5292**/5.3275 ↑
Spin_move4	Mean	6.8641/4.1975	2.8499/4.0148	16.3806/4.4178	14.6455/4.1447	13.4458/4.0248	**2.2562**/4.1285 ↑
	Median	6.097/4.4394	2.786/4.1166	15.6404/4.7137	13.917/4.3189	14.1733/4.0284	**2.3056**/4.5223 ↑
	RMSE	7.8865/4.5753	3.2994/4.3851	17.7753/4.8355	16.3221/4.5232	14.4809/4.4117	**2.4836**/4.5508 ↑
Spin_move5	Mean	**1.7864**/1.8822	**1.3078**/1.9618 ↑	33.2181/1.6030	2.0107/1.7846	**1.6161**/1.8418	2.7025/1.8386
	Median	**1.7204**/1.5188	**1.2788**/1.6052 ↑	26.1658/1.7265	1.9731/1.4896	**1.5320**/1.4320	2.6953/1.4755
	RMSE	**2.1640**/2.3535	**1.5237**/2.4532 ↑	42.2767/1.8329	2.6537/2.2030	**1.9955**/2.3295	3.5095/2.2956
Spin_move6	Mean	13.6722/0.9434	**0.6434**/0.9332 ↑	46.6108/0.9363	**0.7195**/0.9432	**0.8378**/0.9505	0.9842/0.9544
	Median	6.8829/0.9880	**0.4331**/0.9815 ↑	56.8551/0.9817	**0.5068**/0.9932	**0.7124**/0.9787	0.8864/0.9844
	RMSE	21.8973/1.1160	**0.8054**/1.1017 ↑	52.1628/1.1061	**0.8797**/1.1143	**1.0111**/1.1253	1.1772/1.1291
Spin_move7	Mean	2.8939/0.8054	0.9454/0.7967	1411/0.7933	0.8374/0.7884	0.8123/0.8002	1.7947/0.7939
	Median	2.2887/0.9796	1.0159/0.9839	1469/0.9766	0.8556/0.9575	**0.8074**/0.9543 ↑	2.0807/0.9192
	RMSE	3.954/0.9625	1.1267/0.9505	1825/0.9471	0.9918/0.9423	0.9729/0.9583	2.031/0.9520
Spin_move8	Mean	4.2439/0.8824	0.8732/0.8513	329.0178/0.8512	**0.7316**/0.8634 ↑	**0.7832**/0.8788	**0.8017**/0.8844
	Median	2.2936/1.0679	0.9903/1.0039	481.1018/1.0141	**0.8610**/1.0398 ↑	**0.9274**/1.0535	**0.9291**/1.0623
	RMSE	6.586/1.0114	1.0114/0.9790	389.8784/0.9772	**0.8484**/0.9885 ↑	**0.9270**/1.0074	**0.8981**/1.0134
Spin_move9	Mean	5.4395/0.7435	0.7816/0.7302	Fail/0.7288	0.7569/0.7336	0.949/0.7529	1.3903/0.7628
	Median	4.4004/0.8579	0.8878/0.8508	Fail/0.8416	0.8768/0.8333	1.0605/0.8417	1.2123/0.8347
	RMSE	7.086/0.8554	0.8975/0.8357	Fail/0.8369	0.8612/0.8469	1.0783/0.8738	1.6942/0.8881
Spin_move10	Mean	1980/0.7985	0.9958/0.8037	27.7235/0.7860	0.9549/0.7925	2.384/0.8557	24.744/0.8828
	Median	2001/0.9004	1.0436/0.8900	29.3865/0.8791	0.9904/0.9080	2.3141/0.9518	29.8661/0.9789
	RMSE	2527/0.9095	1.1376/0.9086	30.5027/0.9016	1.0575/0.9045	2.8871/0.9694	28.2283/1.0014

### Appendix A.4. Strong Illumination

**Table A6:** The algorithm errors for strong illumination scene with six different MEMS-IMUs.

Strongillumination	ATE	MPU6050	HI219	NV-MG-201	ADIS16488	ADIS16490	MSCRG
Vary_velocity0	Mean	1.4843/1.0077	**0.9915**/1.0183	1.4721/0.9971	**0.9666**/0.9841	**0.7658**/0.9681 ↑	0.9828/0.9524
	Median	1.628/1.1907	**1.1787**/1.2115	1.2117/1.1863	**1.1687**/1.1491	**0.7188**/1.1042 ↑	1.0526/1.0672
	RMSE	1.775/1.1835	**1.1552**/1.1969	1.7483/1.1716	**1.1383**/1.1544	**0.8789**/1.1350 ↑	1.1748/1.1158
Fast0	Mean	**1.0157**/1.1616	**1.0542**/1.1764	**1.1456**/1.1610	**0.8650**/1.1382 ↑	**0.8858**/1.1114	**0.9009**/1.0884
	Median	**1.2064**/1.4117	**1.3031**/1.4534	**1.2621**/1.4259	**1.0067**/1.3778 ↑	**1.0558**/1.3304	**1.1053**/1.2560
	RMSE	**1.2024**/1.3876	**1.2778**/1.4068	**1.4005**/1.3871	**1.0288**/1.3576 ↑	**1.0675**/1.3238	**1.069**/1.2939
Normal0	Mean	**0.8409**/0.9271	**0.8953**/0.9434	**0.9146**/0.9304	**0.7958**/0.9082 ↑	**0.8329**/0.8737	**0.7622**/0.8510
	Median	**1.0407**/1.1921	**0.8647**/1.1856	**1.1182**/1.1789	**0.9343**/1.1743 ↑	**1.0235**/1.0877	**0.8512**/1.0243
	RMSE	**1.0102**/1.1267	**1.0873**/1.1498	**1.1284**/1.1330	**0.9649**/1.0991 ↑	**0.9992**/1.0564	**0.9158**/1.0273
Slow0	Mean	**1.039**/1.1498 ↑	**1.1163**/1.1605	**1.1237**/1.1531	**1.0662**/1.1316	**1.0539**/1.1189	**0.9892**/1.0922
	Median	**1.0382**/1.1812 ↑	**1.1143**/1.1965	**1.1434**/1.1837	**1.0466**/1.1507	**1.0229**/1.1134	**0.9656**/1.0583
	RMSE	**1.2245**/1.3645 ↑	**1.3212**/1.3778	**1.3313**/1.3699	**1.2667**/1.3437	**1.2483**/1.3262	**1.1685**/1.2973
Spin_move0	Mean	111.9958/1.1297	**0.8543**/1.1502 ↑	Fail/1.1275	**0.9878**/1.1192	1.768/1.0893	2.0928/1.0848
	Median	146.6053/1.0838	**0.9553**/1.1028 ↑	Fail/1.0713	**1.0784**/1.0584	1.4484/1.0151	2.2004/0.9645
	RMSE	128.2474/1.3380	**0.9831**/1.3575 ↑	Fail/1.3321	**1.1702**/1.3290	2.2083/1.3028	2.3653/1.3043
Vary_velocity1	Mean	**0.7735**/0.8498	**0.6178**/0.8052 ↑	1.9662/0.8364	0.9341/0.8711	**0.8033**/0.9173	**0.9016**/0.9489
	Median	**0.7705**/0.8905	**0.6200**/0.8349 ↑	2.0385/0.8892	0.8833/0.9074	**0.5831**/0.9699	**0.8170**/1.0091
	RMSE	**0.9017**/1.0073	**0.7082**/0.9524 ↑	2.3543/0.9914	1.058/1.0341	**1.1307**/1.0902	**1.0409**/1.1280
Spin_move1	Mean	14.338/0.8273	**0.5961**/0.8104 ↑	Fail/0.8201	**0.6550**/0.8236	1.1148/0.8598	2.0208/0.8599
	Median	11.3966/0.5462	**0.5879**/0.5573 ↑	Fail/0.5484	**0.6725**/0.5388	1.2885/0.5925	1.4855/0.6288
	RMSE	18.6681/1.0249	**0.6690**/0.9988 ↑	Fail/1.0167	**0.7584**/1.0240	1.3953/1.0637	2.6148/1.0571

## Appendix B

The specific elements of ***F*** and ***V***:

$$F=\left[\begin{array}{ccccc} I & f_{12} & f_{13} & f_{14} & f_{15}\\ 0 & f_{22} & 0 & 0 & f_{25}\\ 0 & f_{32} & I & f_{34} & f_{35}\\ 0 & 0 & 0 & I & 0\\ 0 & 0 & 0 & 0 & I \end{array}\right]V=\left[\begin{array}{cccccc} v_{11} & v_{12} & v_{13} & v_{14} & 0 & 0\\ 0 & v_{22} & 0 & v_{24} & 0 & 0\\ v_{31} & v_{32} & v_{33} & v_{34} & 0 & 0\\ 0 & 0 & 0 & 0 & v_{45} & 0\\ 0 & 0 & 0 & 0 & 0 & v_{56} \end{array}\right]$$

$$\;\;\;\;\;\;\;\;\;\;\;\;\;\;\;\;\;\;\;\;\;\;\;\;f_{12}=\frac{\partial \alpha_{b_{i}b_{k+1}}}{\partial \delta \theta_{b_{k}b_{k}^{\prime }}}=-\frac{1}{4}\left(R_{b_{i}b_{k}}{\left[a^{b_{k}}-b_{k}^{a}\right]}_{\times }\delta t^{2}+R_{b_{i}b_{k+1}}\left[\left(a^{b_{k+1}}-b_{k}^{a}\right)\right]\times \left(I-{\left[w\right]}_{\times }\delta t\right)\delta t^{2}\right)\;$$

$$\;\;\;\;\;f_{13}=\frac{\partial \alpha_{b_{i}b_{k+1}}}{\partial \delta \beta_{b_{k}b_{k}^{\prime }}}=I\delta t$$

$$\;\;\;f_{14}=\frac{\partial \alpha_{b_{i}b_{k+1}}}{\partial \delta b_{k}^{a}}=-\frac{1}{4}\left(q_{b_{i}b_{k}}+q_{b_{i}b_{k+1}}\right)\delta t^{2}$$

$$\;f_{15}=\frac{\partial \alpha_{b_{i}b_{k+1}}}{\partial \delta b_{k}^{g}}=\frac{1}{4}\left(R_{b_{i}b_{k+1}}{\left[a^{b_{k+1}}-b_{k}^{a}\right]}_{\times }\delta t^{2}\right)\delta t$$

$$\;\;\;\;\;\;f_{22}=\frac{\partial \theta_{b_{i}b_{k+1}}}{\partial \delta \theta_{b_{k}b_{k}^{\prime }}}=I-{\left[w\right]}_{\times }$$

$$\;f_{25}=\frac{\partial \theta_{b_{i}b_{k+1}}}{\partial \delta b_{k}^{g}}=-I\delta t$$

$$f_{32}=\frac{\partial \beta_{b_{i}b_{k+1}}}{\partial \delta \theta_{b_{k}b_{k}^{\prime }}}=-\frac{1}{2}\left(R_{b_{i}b_{k}}{\left[a^{b_{k}}-b_{k}^{a}\right]}_{\times }\delta t+R_{b_{i}b_{k+1}}{\left[\left(a^{b_{k+1}}-b_{k}^{a}\right)\right]}_{\times }\left(I\delta t-{\left[w\right]}_{\times }\delta t^{2}\right)\right)$$

$$f_{34}=\frac{\partial \beta_{b_{i}b_{k+1}}}{\partial \delta b_{k}^{a}}=-\frac{1}{2}\left(q_{b_{i}b_{k}}+q_{b_{i}b_{k+1}}\right)\delta t$$

$$f_{35}=\frac{\partial \beta_{b_{i}b_{k+1}}}{\partial \delta b_{k}^{g}}=\frac{1}{2}\left(R_{b_{i}b_{k+1}}{\left[a^{b_{k+1}}-b_{k}^{a}\right]}_{\times }\delta t\right)\delta t$$

$$v_{11}=\frac{\partial \alpha_{b_{i}b_{k+1}}}{\partial n_{k}^{a}}=\frac{1}{4}q_{b_{i}b_{k}}\delta t^{2}$$

$$v_{12}=\frac{\partial \alpha_{b_{i}b_{k+1}}}{\partial n_{k}^{g}}=v_{14}=\frac{\partial \alpha_{b_{i}b_{k+1}}}{\partial n_{k+1}^{g}}=-\frac{1}{8}\left(R_{b_{i}b_{k+1}}{\left[a^{b_{k+1}}-b_{k}^{a}\right]}_{\times }\delta t^{2}\right)\delta t$$

$$v_{13}=\frac{\partial \alpha_{b_{i}b_{k+1}}}{\partial n_{k+1}^{a}}=\frac{1}{4}q_{b_{i}b_{k+1}}\delta t^{2}$$

$$v_{22}=\frac{\partial \theta_{b_{i}b_{k+1}}}{\partial n_{k}^{g}}=v_{24}=\frac{\partial \theta_{b_{i}b_{k+1}}}{\partial n_{k+1}^{g}}=\frac{1}{2}I\delta t$$

$$v_{31}=\frac{\partial \beta_{b_{i}b_{k+1}}}{\partial n_{k}^{a}}=\frac{1}{2}q_{b_{i}b_{k}}\delta t$$

$$v_{33}=\frac{\partial \beta_{b_{i}b_{k+1}}}{\partial n_{k+1}^{a}}=\frac{1}{2}q_{b_{i}b_{k+1}}\delta t$$

$$v_{32}=\frac{\partial \beta_{b_{i}b_{k+1}}}{\partial n_{k}^{g}}=v_{34}=\frac{\partial \beta_{b_{i}b_{k+1}}}{\partial n_{k+1}^{g}}=-\frac{1}{4}\left(R_{b_{i}b_{k+1}}{\left[a^{b_{k+1}}-b_{k}^{a}\right]}_{\times }\delta t^{2}\right)\delta t$$

$$v_{45}=\frac{\partial b_{k+1}^{a}}{\partial n_{b_{k}^{a}}}=v_{56}=\frac{\partial b_{k+1}^{g}}{\partial n_{b_{k}^{g}}}=I\delta t$$
